# Overcrowding in Poor Law Infirmaries

**Published:** 1912-03-30

**Authors:** 


### OVERCROWDING IN POOR LAW INFIRMARIES.

### The Medical Officer on the Position at Worcester.

The condition of overcrowding at many Poor Law infirmaries to-day, and the general reluctance of the Guardians throughout the country to undertake any form of building, has made it a matter of importance for The Hospital's readers to understand the true condition of affairs. With this end in view our Commissioner paid a special visit to the medical officer of the Worcester Workhouse Infirmary, Mr. S. Legge, to discover from him the nature of the reform scheme for providing additional accommodation at this Poor Law Infirmary, the meeting at which the new scheme was discussed having been alluded to in The Hospital.

On being asked whether overcrowding had become a chronic feature at the Worcester Poor Law Infirmary, Mr. Legge said :

### Some Causes of Overcrowding.

"I regret to say it has, partly because this infirmary, like many others, has to provide many beds for aged persons, whose condition often borders upon senile decay, or who have virtually become mental cases. Again, though the workhouse, as you will see in a moment, has been relieved to a certain extent by the operation of the Old Age Pensions Act, nevertheless in many cases those in receipt of pensions find that they cannot live upon them, and drift or prefer to return to the workhouse proper. So it has not been a complete gain, even here; and in the case of the infirmary the number of phthisical patients, very often advanced cases, tends to increase. I regret to say that many of these patients become "in and outers"—that is, they frequently improve (up to a certain point), take their discharge, and sooner or later come in again, in the meantime doubtless having infected dwelling-places and those inhabiting them. If this could be prevented the gain to the community would be great, as it is the advanced cases which spread the disease by spitting, etc., not those in the early stage, who only are admitted to sanatoria.

"Then as to the additional accommodation scheme?"

"In the light of what I have said already, you will see that here, as elsewhere, the problem of overcrowding has become increasingly urgent, and here, as elsewhere, there has been more than one scheme of relief proposed. I was asked by the Guardians to draw up a report not long ago, and I took this latest opportunity of adding the suggestion that two (additional) open-air pavilions for phthisical cases should be built in the grounds. The Guardians, however, among other reasons, were chary of incurring the necessary expense and of once more raising the rates. As The Hospital has already noted, at the meeting which rejected my scheme by eighteen votes, a member suggested that the objectors should thereupon resolve themselves into a committee to draw up an alternative proposal. It was something of a Gilbertian situation, but, like many Gilbertian situations, led to unexpected results. The objectors, who, broadly speaking, were in favour of adapting the existing buildings rather than erecting new ones, discovered that the overcrowding, which all admitted to prevail in the infirmary, did not prevail in the female wards of the workhouse. This led them to propose that these empty dormitories should be adapted to relieve the congestion. Another interesting point is that one objection raised against my scheme was that it was undesirable for the town to have wards erected for phthisical patients in its midst, the idea evidently being that tuberculosis is as infectious as small-pox, typhus, etc. I did not agree with this contention, nor do I now see how matters will be improved when the phthisical patients, instead of being housed in their own pavilion, will still be housed in the workhouse itself. On the other hand, one can understand the Guardians' unwillingness to build phthisical wards, in so far as it is influenced by the present proposals of the Government to erect at some indefinite date sanatoria or some kindred institutions all over the country."

"How will the Guardians' scheme affect administration?"

"There, of course, a certain difficulty is bound to arise. A staff endeavouring to work two buildings detached from one another is never so easy or so possible to manage as one. This did not apply against my scheme, as the infirmary would have been connected with the pavilion by a covered way. But the wards in the workhouse cannot be so connected with the infirmary. I made at once the stipulation that the present nursing staff should not be regarded as sufficient for the additional work, and I am urging the importance of increasing it by two nurses, whose sole work will be in the adapted wards. This recommendation has been rejected in favour of additional nurses being engaged when necessary."

### The General Moral.

"How long will it be before the adapted quarters are ready?"

"Certain alterations to windows and so forth have to be made, together with the reduction of the present number of beds, which, though more or less allowable when their occupiers are healthy, do not provide sufficient cubic space of air to comply with the Government regulations for the sick. You will now be able to contrast the two schemes with each other, to compare the above summaries, which is perhaps worth a little study because it illustrates a position which, in slightly divergent forms, exists throughout the Poor Law infirmaries of the country. On the one hand, in short, critics of the Guardians will be found to say that dilatory methods and the multiplication of reports and committees are not infrequently found, while on the other it must be frankly admitted that the present is not a time when buildings can be undertaken lightly by men who realise the uncertainties of legislation, and who do not want to raise the rates more than they can help."

By the courtesy of the master of the workhouse, Mr. Roberts, our Commissioner was shown over the Poor Law Infirmary and the workhouse, and was thus able to inspect the wards which are to be adapted, and to see the site on which the new pavilions, had Mr. Legge's scheme been followed, would have been built.

